# Polyethylene Glycol-*b*-poly(trialkylsilyl methacrylate-*co*-methyl methacrylate) Hydrolyzable Block Copolymers for Eco-Friendly Self-Polishing Marine Coatings

**DOI:** 10.3390/polym14214589

**Published:** 2022-10-28

**Authors:** Elisa Guazzelli, Matteo Oliva, Carlo Pretti, Gianfranca Monni, Armand Fahs, Christine Bressy, Elisa Martinelli

**Affiliations:** 1 Dipartimento di Chimica e Chimica Industriale, Università di Pisa, 56124 Pisa, Italy; 2 Consorzio Interuniversitario di Biologia Marina e Ecologia Applicata “G. Bacci”, 57128 Livorno, Italy; 3 Dipartimento di Scienze Veterinarie, Università di Pisa, 56124 Pisa, Italy; 4 Laboratoire MAPIEM, Université de Toulon, E.U.4323, SeaTech Ecole d’Ingénieurs, CS 60584, CEDEX 9, 83041 Toulon, France

**Keywords:** amphiphilic polymer, hydrolyzable polymer, trialkylsilyl methacrylate, self-polishing coatings, polyethylene glycol

## Abstract

Hydrolyzable block copolymers consisting of a polyethylene glycol (PEG) first block and a random poly(trialkylsilyl methacrylate (TRSiMA, R = butyl, isopropyl)-*co*-methyl methacrylate (MMA)) second block were synthesized by RAFT polymerization. Two PEGs with different molar masses (*M*_n_ = 750 g/mol (PEG1) and 2200 g/mol (PEG2)) were used as macro-chain transfer agents and the polymerization conditions were set in order to obtain copolymers with a comparable mole content of trialkylsilyl methacrylate (~30 mole%) and two different PEG mole percentages of 10 and 30 mole%. The hydrolysis rates of PEG-*b*-(TRSiMA-*co*-MMA) in a THF/basic (pH = 10) water solution were shown to drastically depend on the nature of the trialkylsilyl groups and the mole content of the PEG block. Films of selected copolymers were also found to undergo hydrolysis in artificial seawater (ASW), with tunable erosion kinetics that were modulated by varying the copolymer design. Measurements of the advancing and receding contact angles of water as a function of the immersion time in the ASW confirmed the ability of the copolymer film surfaces to respond to the water environment as a result of two different mechanisms: (i) the hydrolysis of the silylester groups that prevailed in TBSiMA-based copolymers; and (ii) a major surface exposure of hydrophilic PEG chains that was predominant for TPSiMA-based copolymers. AFM analysis revealed that the surface nano-roughness increased upon immersion in ASW. The erosion of copolymer film surfaces resulted in a self-polishing, antifouling behavior against the diatom *Navicula salinicola*. The amount of settled diatoms depended on the hydrolysis rate of the copolymers.

## 1. Introduction

After the international ban of tributyltin (TBT)-based self-polishing coatings (SPCs) in 2008, the two most effective antifouling (AF) technologies actually present on the market for the marine shipping industries are nontoxic fouling-release coatings (FRCs) which are able to promote the detachment of eventually settled foulers under the action of relatively low shear stresses, and self-polishing coatings (SPCs), which act on marine organisms by inhibiting or limiting their settlement using the release of biocides embedded or linked to a polymer matrix [1,2,3,4,5,6]. The former coatings are mainly based on poly(dimethylsiloxane) (PDMS) elastomer matrixes containing amphiphilic surface-active (co)polymers [7,8,9,10,11], and as a result of their surface segregation promote the hydrophilization of the PDMS surface, thus enhancing its AF performance in static conditions and against microfouling [11,12,13,14,15,16]. These coatings have been widely investigated [9,17,18,19] and currently have a modest market share, especially because of the higher cost and lower efficiency of static conditions compared with traditional SPCs. On the other hand, self-polishing coatings (SPCs) that contain copper oxide (Cu_2_O) as biocide currently dominate the market, and base their AF activity on the presence of seawater hydrolyzable (meth)acrylate groups that lead to the renewal of the coating surface by an erosion process, with a simultaneous release of biocide copper species with a controlled rate. Such SPCs generally also contain booster biocides (2.5–10 wt%) that enhance the AF activity of the metal-based biocide against target organisms and, in some cases, non-target organisms as well [20,21,22,23,24,25]. Nowadays, these coatings are becoming progressively more regulated because of environmental concerns due to their high level of copper leaching, negatively affecting different marine organisms at various stages of their life. There is, therefore, a growing interest in developing nontoxic, biocide-free AF coatings that operate with a self-polishing mechanism [26,27,28,29]. One valid alternative reported in the literature is the use of trialkylsilyl(meth)acrylate-based (co)polymers as binders for SPCs [30,31,32,33,34,35,36], as they are able to be eroded in seawater through the hydrolysis of the silyl-ester bond. However, these systems generally contain metal-based and/or booster biocides, though in a reduced amount [37,38,39,40,41,42,43]. In a more eco-friendly approach [44,45], amphiphilic hydrolyzable block copolymers composed of a poly(dimethylsiloxane) (PDMS) first block and a random poly(ethylene glycol) methacrylate (PEGMA)-*co*-trialkylsilyl methacrylate second block are used as surface-active additives in a PDMS matrix to obtain biocide-free SP/FR hybrid systems, in which the copolymer hydrolysis rate can be modulated by varying the amount of PEG in the copolymer.

In this study, novel amphiphilic diblock copolymers PEG-*b*-(TRSiMA-*co*-MMA) were synthesized via RAFT polymerization. PEG macromolecular RAFT chain transfer agents of two different lengths were used as macroinitiators for the copolymerization of the second random block composed of a methyl methacrylate (MMA) and a hydrolyzable trialkylsilyl methacrylate (TRSiMA, R = butyl or isopropyl). In the copolymer design, the amount of TRSiMA was ~30 mole% to appreciate the hydrolysis process, while that of MMA was higher than ~40 mole% to obtain self-supporting films directly from the copolymers without the use of an additional matrix. The copolymer hydrolysis kinetics was investigated and the effects of the type of trialkylsilyl ester and the content of PEG and MMA were evaluated. Films derived from selected copolymers were characterized by surface erosion measurements, dynamic contact angles and AFM analyses, and structure–property correlations were highlighted. Biological performance was evaluated against the diatom *Navicula salinicola* chosen as the model microorganism, because slimes dominated by diatoms are the predominant form of microfouling against which the antifouling efficiency of fouling-release PDMS-based coatings are known to be poor [14,15,46].

## 2. Materials and Methods

### 2.1. Materials

Dichloromethane (DCM) and toluene (Sigma Aldrich, Darmstadt, Germany) were distilled over CaH_2_ before use. 4-Cyano-4-[(dodecylsulfanylthiocarbonyl)sulfanyl]pentanoic acid (CTA), *N*,*N*′ dicyclohexylcarbodiimide (DCC), and 4-(dimethylamino) pyridine (DMAP) were purchased from Sigma Aldrich and used as received. 2,2′-Azobis(isobutyronitrile) (AIBN, Sigma Aldrich, Darmstadt, Germany) was recrystallized from methanol. Polyethylene glycols monomethyl ether (Sigma Aldrich, Darmstadt, Germany, PEG1 (*M*_n_ = 750 g/mol) and PEG2 (*M*_n_ = 2000 g/mol)) were dissolved in toluene; then, water was removed by azeotropic distillation and finally the dried polymer was stored under nitrogen. Methyl methacrylate (MMA, Sigma Aldrich, Darmstadt, Germany) was distilled under reduced pressure to remove polymerization inhibitors. Tributyl silyl methacrylate (TBSiMA, Yuki Gosei Kogyo Co., Tokyo, Japan) and tri-isopropylsilyl methacrylate (TPSiMA, Yuki Gosei Kogyo Co., Ltd., Tokyo, Japan) were dissolved in dichloromethane and passed through a column of neutral alumina to remove inhibitors. Common laboratory solvents and other reagents (Sigma Aldrich, Darmstadt, Germany) such as chloroform, chloroform-d, acetone, diethyl ether, n-hexane, dichloromethane, methanol, tetrahydrofuran and tetrahydrofuran-d8 were used as received. Sea salt (Aldrich, Darmstadt, Germany) was used to prepare an artificial seawater with a salinity of 36 g/L.

### 2.2. Characterizations

^1^H NMR measurements were carried out on a Bruker Avance 400 MHz spectrometer with deuterated solvents at room temperature. The sample concentration was approximately 20–25 g/L. The hydrolysis kinetics of the silylester bond of TRSiMA units in solution was monitored by ^1^H NMR spectrometry at different times. All samples (10 mg) were dissolved in 750 μL of THF-d8 to which 100 μL of a sodium bicarbonate buffer solution (pH = 10) was added.

The number and weight average molecular weights (*M*_n_, *M*_w_) were determined by gel permeation chromatography (GPC) using a Jasco (Tokyo, Japan) PU-2089Plus liquid chromatograph pump equipped with two PL gel 5 μm mixed-D columns, a Jasco RI- 2031 Plus refractive index detector, and a Jasco UV-2077Plus UV/vis detector. Measurements were carried out using chloroform as the mobile phase at a flux of 1 mL/min and a temperature of 30 °C maintained by a Jasco CO 2063 Plus column thermostat. Polymethyl methacrylate standards were used for calibration. Samples were prepared at 5 mg/mL and they were filtered through a 0.2 μm PTFE filter before injection.

Dynamic contact angle (DCA) experiments were carried out by the advancing–receding drop method using a DSA 30 apparatus (Krüss, Hambourg, Germany) under ambient conditions. A 10 μL deionized water drop was first placed onto the coating with the syringe tip still immersed within the droplet; then, the droplet was grown at a rate of 0.75 μL/s until a final volume of 25 μL was reached for the measurement of the advancing contact angle (*θ*_adv_). The receding contact angle (*θ*_rec_) was measured by withdrawing the liquid at the same rate. It has to be specified that the syringe tip was placed as close as possible to the coating surface to avoid any droplet distortion when aspirating the liquid. For each coating, the reported *θ*_adv_ and *θ*_rec_ were the average values of at least three droplets.

Atomic force microscopy (AFM) experiments were performed using a Multimode 8 microscope (Bruker, Billerica, MA, USA) in PeakForce Quantitative Nanomechanics mode (PF-QNM). The measurements were carried out in air at ambient conditions. The samples were rinsed and dried in air to avoid any contamination of the AFM probe before being tested. The silicon probe was selected based on the recommendation of the AFM producer (RTESPA-300). The spring constant of the cantilever was measured using the Sader method and was found to be 33.8 N m^−1^ [47]. The AFM probe was calibrated on a polystyrene reference sample, and the surface elastic modulus of the films was calculated using a Derjaguin–Muller–Toropov (DMT) contact mechanics model [48]. AFM images were collected on 3 different regions of the films with a resolution of 256 × 256 pixels and a scan rate of 0.8 Hz.

### 2.3. Synthesis

#### 2.3.1. Synthesis of the Macroinitiators PEGz-CTA

The synthesis of PEG2-CTA is described as an example. Amounts of 0.901 g of CTA (2.25 mmol), 0.055 g of DMAP (0.45 mmol) and 10 mL of anhydrous dichloromethane were loaded into a 100 mL three-neck round-bottom flask equipped with a magnetic stir bar, condenser and two dropping funnels under a nitrogen atmosphere. Then, 3 g of PEG2 (1.5 mmol) and 0.464 g of DCC (2.25 mmol) were dissolved in 10 and 20 mL of dry DCM, respectively, introduced in the dropping funnels, and then added dropwise into the flask maintaining vigorous stirring. The flask was first placed in an ice bath, then allowed to warm to room temperature under stirring for 24 h. The reaction mixture was first filtered to remove the solids and, subsequently, the polymer was precipitated two times into *n*-hexane to remove additional residual DCU and the excess of CTA (yield 85%). The pure product was characterized by ^1^H NMR (Figure S1).

^1^H NMR (CDCl_3_, δ in ppm): 4.27 (COOC*H*_2_), 3.9–3.5 (CH_2_C*H*_2_O), 3.4 (OC*H*_3_), 3.35 (SC*H*_2_), 2.7 (C*H*_2_COO), 2.3–2.6 (C*H*_2_CH_2_COO), 1.9 (C*H*_3_CCN), 1.7 (SCH_2_C*H*_2_), 1.5–1.2 (C*H*_2_)_9_, 0.9 (CH_2_C*H*_3_).

#### 2.3.2. Synthesis of PEGz-*b*-(TRSiMAx-*co*-MMAy) Copolymers

The synthesis of PEG2-*b*-(TBSiMA26-*co*-MMA41) and PEG2-*b*-(TPSiMA28-*co*-MMA38) will be described as examples of the two series of copolymers. For PEG2-*b*-(TBSiMA26-*co*-MMA41), AIBN (0.0028 g, 0.017 mmol), PEG2-CTA (0.223 g, 0.086 mmol), TBSiMA (1.232 g, 4.331 mmol), MMA (0.578 g, 5.775 mmol), and anhydrous toluene (6.7 mL) were charged into a 100 mL Carius tube with a magnetic stirrer bar. The reaction mixture was then degassed by three freeze–pump–thaw cycles and then sealed under vacuum. The polymerization was carried out at 70 °C in an oil bath under magnetic stirring for 72 h. The reaction was stopped by exposure to air and cooled down to room temperature. The polymer was purified by three precipitations from DCM solutions into a large excess of methanol (yield 40%). The obtained copolymer, named PEG2-(TBSiMA26-*co*-MMA41), contained 26 mole% TBSiMA, 41 mole% MMA and 33 mole% PEG2 co-units. The pure product was characterized by ^1^H NMR (Figure S2).

GPC (CHCl_3_): *M*_n_ = 12,700 g/mol; *Ð* = 1.1

^1^H NMR (CDCl_3_, δ in ppm): 4.27 (COOC*H*_2_), 3.8–3.5 (OC*H*_2_C*H*_2_O, C*H*_3_OOC), 3.4 (CH_2_OC*H*_3_), 2.2–1.6 (C*H*_2_C(CH_3_)), 1.5–1.2 (SiCH_2_C*H*_2_C*H*_2_CH_3_), 1.2–0.7 (CH_2_C(C*H*_3_); SiCH_2_CH_2_CH_2_C*H*_3_, SiC*H*_2_CH_2_CH_2_CH_3_).

For PEG2-*b-*(TPSiMA28-*co*-MMA38), AIBN (0.0031 g, 0.019 mmol), PEG2-CTA (0.245 g, 0.095 mmol), TPSiMA (1.155 g, 4.764 mmol) and MMA (0.636 g, 6.360 mmol) in anhydrous toluene (7.4 mL) were charged into a 100 mL Carius tube along with a magnetic stirrer bar. The reaction mixture was then degassed by three freeze–pump–thaw cycles and sealed under vacuum. The reaction was carried out at 70 °C for 90 h. The polymer was purified by three precipitations in *n*-hexane from DCM solutions (yield = 35%). The obtained copolymer, named PEG2-*b-*(TPSiMA28-*co*-MMA38), contained 34 mol% PEG2, 28 mole% TPSiMA and 38 mole% MMA co-units. The pure product was characterized by ^1^H NMR (Figure S3).

GPC (CHCl_3_) *M*_n_ = 14,700 g/mol; *Ð* = 1.1

^1^H NMR (CDCl_3_, δ in ppm): 4.27 (COOC*H*_2_), 3.8–3.5 (OC*H*_2_C*H*_2_O; C*H*_3_OOC), 3.4 (CH_2_OC*H*_3_), 2.3–1.6 (C*H*_2_C(CH_3_)), 1.6–0.7 (CH_2_C(C*H*_3_), SiCH(C*H*_3_)_2_, SiC*H*(CH_3_)_2_).

Random copolymers TBSiMAx-*co*-MMAy and TPSiMAx-*co*-MMAy were also synthesized by RAFT polymerization as reference samples.

### 2.4. Film Preparation

Copolymer films for mass erosion, contact angle and AFM analyses were prepared on 2.5 × 1.5 cm^2^ or 1 cm diameter sandblasted PVC panels and foils by the solution casting of 35 wt% solution in xylene. A bar coater was used to control the thickness of the films. The solvent was allowed to evaporate slowly at room temperature. The final dry thickness of the films was ~100 μm. Copolymer films for biological assays against marine organisms were prepared by casting on 7.5 × 2.5 cm^2^ sandblasted PVC panels with 13 wt% copolymer solution in xylene. The solvent was allowed to evaporate slowly at room temperature. The final dry thickness of the films was ~100 μm.

Studies of mass erosion were carried out according to a procedure reported in the literature [49]. Briefly, the mass loss of coatings was monitored gravimetrically during static immersion tests in artificial seawater at room temperature. Periodically, the panels extracted from the water were rinsed with deionized water, dried for 24h in air, and weighed. The mass variation (%) was calculated from Equation (1):

(1) $$Mass\;variation\;\left(\%\right)=100\;\frac{w_{t}-w_{0}}{w_{0}-w_{panel}}$$

where *w_t_*, *w*_0_ and *w_panel_* are the weight of the coated PVC foil at time *t*, at time *t* = 0, and the weight of the initial non-coated PVC foil, respectively.

### 2.5. Biological Assay

All the films were equilibrated in filtered natural seawater (membrane pore size 0.45 µm, salinity 30 psu) for 24 h prior to testing.

Algal stock suspension of *Navicula salinicola* was prepared by adding 50 mL of *N. salinicola* laboratory axenic strain (maintained at the Ecotoxicology and Microbiology laboratory of CIBM, Livorno, Italy) to 500 mL of fresh sterile *F*/2 + Si medium 72 h before the experimental setup. The new suspension was maintained at 20 ± 2 °C under continuous illumination (light intensity 6000 lux) to assure logarithmic algal growth during the experiment. Algal biomass quantification, in terms of chlorophyll (A + C) content, was obtained by measuring the autofluorescence intensity of several known algal concentrations at 435 nm as excitation wavelength and 682 nm as emission wavelength, and calculating a regression line equation with a microplate reader (Synergy-HTX Biotech, Agilent, CA, USA). Before the experimental exposure of films to the algal suspension, the intrinsic fluorescence emission of all coated slides was assessed as background noise, using the same protocol for chlorophyll autofluorescence. Adhesion of the diatom *N. salinicola* was assessed by exposing films to a 10^4^ cell/mL algal suspension (~10 mL) using 4-well Quadriperm^®^ plates. Plates were incubated at 20 ± 2 °C with alternating light for 24 h (14 h of light and 10 h of darkness, light intensity 3000 lux) under continuous gentle agitation (~80 rpm). After this period, all films were gently rinsed with fresh filtered seawater and adherent algal biomass was evaluated, as described above, by converting the fluorescence reading—corrected for background noise in the algal biomass—with the previously calculated equation. The procedure was repeated for different exposure times of 48 h, 72 h, 6 and 9 days following the same procedure, exposing films again to the algal suspension for the requested additional time and evaluating the algal biomass at each time step.

## 3. Results and Discussion

### 3.1. Synthesis of Copolymers

**Figure 1:**
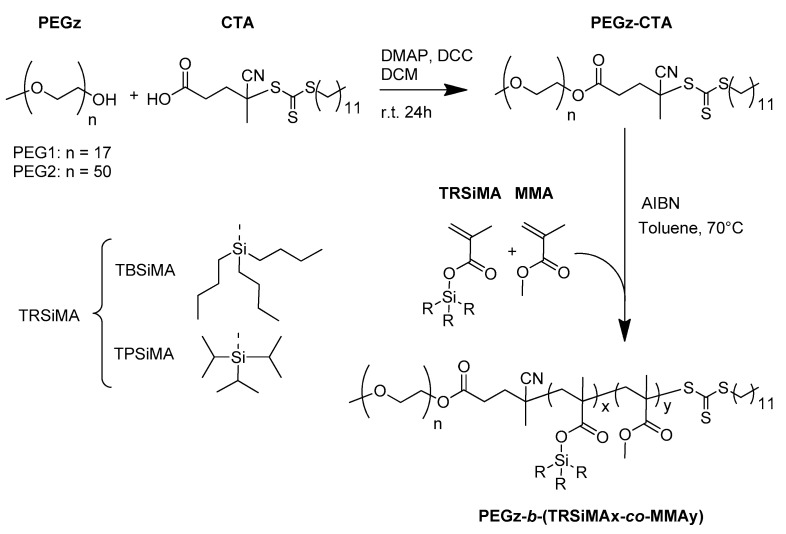
Synthesis of amphiphilic hydrolyzable diblock copolymers via RAFT copolymerization of TRSiMA (R = isopropyl, butyl) and MMA with PEGz-CTA.

Amphiphilic hydrolyzable block copolymers of PEG-*b*-(TRSiMA-*co*-MMA) were synthesized starting from a PEG-based chain transfer agent, prepared by adapting a literature procedure [34,45]. They were synthesized via the Steglich esterification of a 4-cyano-4-(dodecyl sulfanyl thiocarbonyl)sulfanyl pentanoic acid (CTA) RAFT chain transfer agent with commercial available monomethoxy and terminated PEGz of different molecular weights (PEG1 *M*_n_ = 750 g/mol, PEG2 *M*_n_ = 2200 g/mol) (Figure 1). This PEGz-CTA was then used for the RAFT polymerization of TBSiMA and TPSiMA leading to two novel series of diblock copolymers (Figure 1). Monomer concentrations and molar ratios of PEGz-CTA:AIBN were fixed at 1.5 M and 5:1, respectively, and the reaction was carried out in anhydrous toluene at 70 °C for 90 h and 72 h in the case of TPSiMA and TBSiMA, respectively, in order to increase the TPSiMA conversion given its high steric hindrance and slower reactivity [45]. The series of TRSiMA-based copolymers were prepared by fixing at 30 mole% the amount of TRSiMA in the feed, varying MMA content to obtain copolymers with ~10 mole% or ~30 mole% of PEG co-units. In such a copolymer design, the hydrophilic PEG first block is, in fact, expected to modulate the hydrolysis rate of the copolymer on the basis of its length and content, while the MMA is incorporated in the second random block to increase the rigidity of the second block and allow the preparation of self-supporting films at room temperature. However, it is also anticipated to affect the hydrolysis kinetics.

Relevant reaction conditions are reported in Table 1, along with the physical–chemical characterization of the products. Amphiphilic block copolymers were named PEGz-*b*-(TRSiMAx-*co*-MMAy), where *x* and *y* refer to the mole percentage of the TRSiMA and MMA repeating units, respectively, in the copolymer.

Copolymer composition and the degree of polymerization (DP_n_) of each component were estimated by ^1^H NMR analysis. Details for calculation are reported in the Supplementary Materials. The calculated composition and DP_n_ are reported in Table 1.

**Figure 2:**
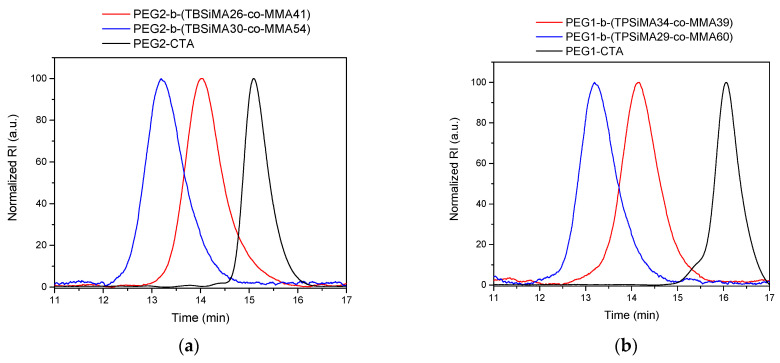
Normalized GPC curves in CHCl_3_ of (**a**) PEGz-*b*-(TBSiMAx-*co*-MMAy) and (**b**) PEGz-*b*-(TPSiMAx-*co*-MMAy) and the corresponding PEG-based macromolecular RAFT chain transfer agents (PEGz-CTA).

Purified copolymers were analyzed via GPC in chloroform that displayed monomodal peaks (Figure 2), corresponding to narrow molar mass dispersities *Ð* between 1.1 and 1.4 (Table 1).

### 3.2. Hydrolysis Kinetics of Copolymers

**Figure 3:**
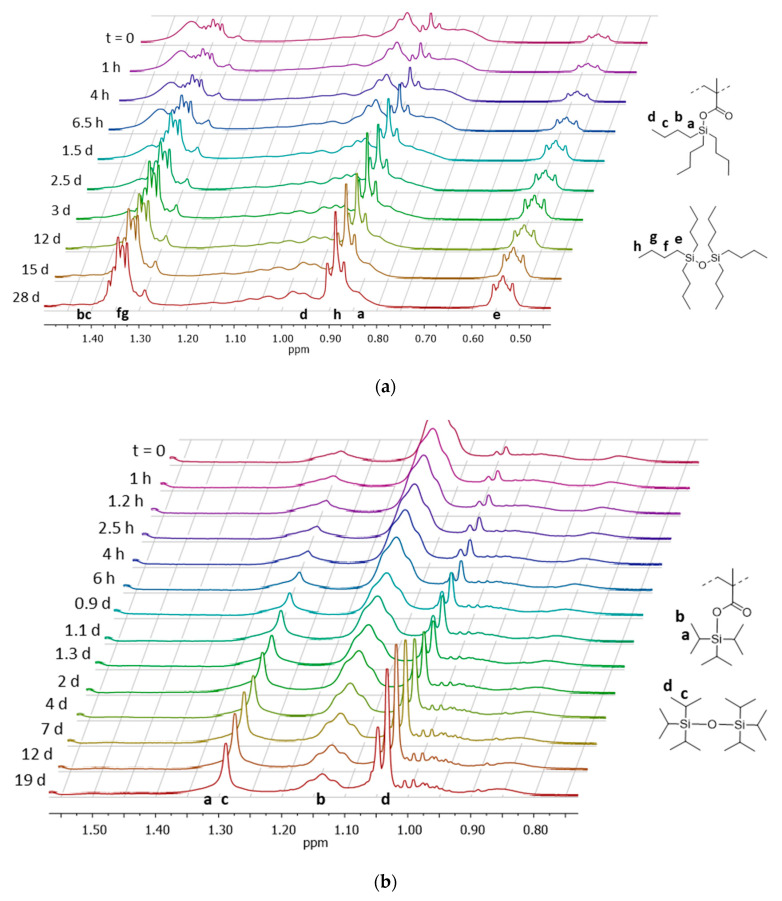
^1^H NMR spectra of the hydrolysis of PEG1-*b*-(TBSiMA24-*co*-MMA49) (**a**) and PEG2-*b*-(TPSiMA28-*co*-MMA38) (**b**) in solution of THF-d8 with pH 10 buffer at different sampling times (increasing from back to front, from *t* = 0 to *t* = 19 or 28 days, respectively).

The hydrolysis rate of some copolymers, representative of each series, was studied in accelerated conditions via in situ ^1^H NMR by dissolving the copolymer in THF-d8 and adding a small aliquot of a pH 10 NaOH/NaHCO_3_ buffer to the solution. The incorporation of a hydrophilic and/or hydrophobic component into a silyl ester-based polymer is expected to modulate hydrolysis kinetics, affecting initial polymer water affinity and water diffusion to the cleavable bonds. ^1^H NMR spectra for the hydrolysis of PEG2-*b*-(TPSiMA28-*co*-MMA38) and PEG1-*b*-(TBSiMA24-*co*-MMA49) are shown in Figure 3. In both cases, the signals of protons of the alkyl substituents of the silylester side groups in the repeating units shifted to lower ppm, passing to the hydrolyzed siloxane product. For example, in case of TBSiMA, the signal of the SiCH_2_ protons in the butyl chain are found at 0.90 ppm in the copolymer but move to 0.55 ppm in the hydrolyzed siloxane product. In a similar manner, methyl protons in the isopropyl group change from 1.15 ppm to 1.05 ppm in the copolymer and the hydrolysis product, respectively. These changes were exploited to evaluate the hydrolysis degree of the copolymers at different times and the calculation details are reported in Supplementary materials.

**Figure 4:**
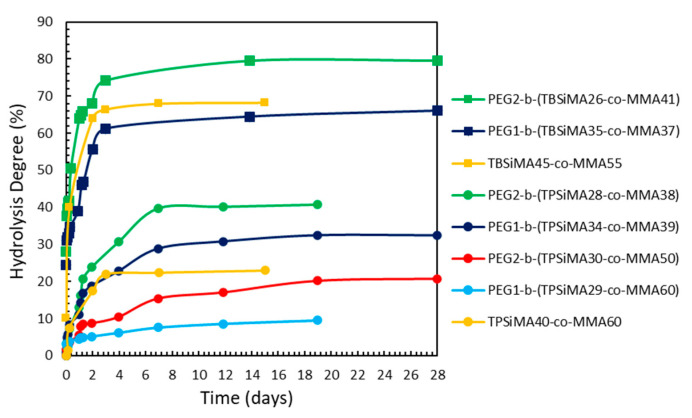
Hydrolysis degree of TBSiMA- and TPSiMA-based copolymers in THF-d8 with pH 10 buffer.

As shown in Figure 4, the hydrolysis profiles were markedly different for the two series of copolymers based on the different TRSiMA units, with slower, incomplete hydrolysis for the TPSiMA-based copolymers (Figure 4) that reached a plateau after about 8 days (with a hydrolysis degree in the range 6–40%). On the other hand, TBSiMA-based copolymers (Figure 4) showed a hydrolysis degree higher than ~60% after only 4 days before reaching the plateau. This behavior is consistent with what was expected from the trends of the corresponding TRSiMA homopolymers [45,50] and the different steric hindrances of the alkyl groups on the silylester. The presence of the PEG block in the amphiphilic copolymers provided different effects. Observing the hydrolysis profiles for the PEGz-*b*-(TPSiMA-*co*-MMA) series, it was found that a higher molar content of EG units in the copolymer (27–34 mole%) was necessary to increase the hydrolysis rate of the sample with respect to the TPSiMA40-*co*-MMA60 copolymer, not containing PEG. Moreover, the longer PEG block in PEG2-*b*-(TPSiMA-*co*-MMA) helped the copolymer to reach higher hydrolysis degrees. On the contrary, when the MMA mole% was higher than 50%, the hydrolysis rate was drastically reduced even in the presence of the PEG block. This is in agreement with what has previously been found for PDMS-*b*-(PEGMA-*co*-TRSiMA) [45], where the addition of hydrophobic components such as PDMS was able to slow down the hydrolysis of the copolymers even in the presence of the PEGMA hydrophilic comonomer. In the case of the PEGz-*b*-(TBSiMA-*co*-MMA) series, we found that the longer PEG block in the PEG2-*b*-(TBSiMA26-*co*-MMA41) copolymer allowed a higher hydrolysis degree than the reference TBSiMA45-*co*-MMA55 and the copolymer PEG1-*b*-(TBSiMA35-*co*-MMA37) characterized by a similar composition, but with a shorter length of the PEG block. Although explicative of the effects of the different structural parameters (i.e., the type of silylester group, incorporation of PEG, and its length), such a study has to be considered only indicative of the erosion rate of copolymer films in a seawater environment. Thus, for a more comprehensive characterization, selected copolymers were used to prepare films and subject them to mass loss evaluation during static immersion in ASW. Moreover, the wettability of the same films was analyzed by dynamic contact angle as a function of immersion in ASW. In particular, PEG2-*b*-(TPSiMA30-*co*-MMA50) was chosen as representative of copolymers with slower hydrolysis rates. This copolymer was paired with PEG2-*b*-(TPSiMA28-*co*-MMA38), which showed an enhanced hydrolysis rate with the effect of the higher PEG content. Finally, PEG2-*b*-(TBSiMA26-*co*-MMA41) was also included as having TBSiMA in place of TPSiMA, and as such being representative of copolymers with higher hydrolysis rates.

### 3.3. Mass Erosion of Copolymer Films in Artificial Seawater

**Figure 5:**
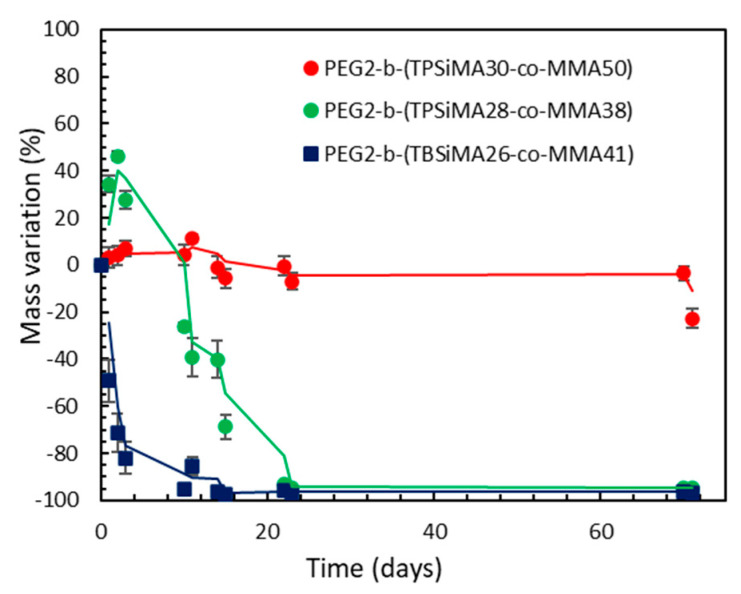
Mass variation of the copolymer films during immersion in artificial seawater. Solid lines are moving average (period n = 2) reported as a guideline. Error bars are the relative error of the weighed sample average from 3 replicates.

The mass variation in the selected copolymer films upon immersion in ASW for 72 days is reported in Figure 5. For PEG2-*b*-(TPSiMA28-*co*-MMA38) a significant gain of mass during the initial immersion period was observed. This was attributed to the preferential absorption of water in the first 4 days that was more remarkable than the surface erosion, as copolymer hydrolysis was still slow due to the higher stability of TPSiMA units. However, on day ten, the hydrolysis of the polymer prevailed over swelling and a mass loss was clearly measured. The diffusion of water within the coating resulted in the complete hydrolysis of the TPSiMA ester side groups within 23 days. On the other hand, PEG2-*b*-(TPSiMA30-*co*-MMA50), with a lower content of PEG (20 mole%) and a reduced hydrophilic character, presented a significantly lower water absorption and a slower erosion of the film that showed only a 23% mass loss after 72 days. Finally, for PEG2-*b*-(TBSiMA26-*co*-MMA41) containing TBSiMA units, hydrolysis prevailed over water absorption. Therefore, the mass decreased continuously up to the complete film erosion after 14 days.

### 3.4. Dynamic Contact Angle Analysis

Dynamic contact angle was measured on selected films at fixed time intervals of 21, 30 and 75 days of immersion in ASW. Results are collected in Table 2. Surfaces of the dry films of PEG2-*b*-(TPSiMA28-*co*-MMA38) and PEG2-*b*-(TBSiMA26-*co*-MMA41) were moderately hydrophilic (*θ*_adv_ = 71°–82° < 90°) as a result of the higher content of PEG units (33−34 mole%). Differently, the PEG2-*b*-(TPSiMA30-*co*-MMA50) dry films with the lowest PEG percentage (20 mole%) showed an exceptionally high *θ*_adv_ (110°), suggesting that in this case the surface was mainly populated by the pendant isopropyl groups of the not-hydrolyzed silylester units that can generate a hydrophobic surface. This is also consistent with the results obtained from the film mass loss experiments, where this sample showed a much slower absorption of water. *θ*_adv_ values of films after immersion in water showed two different trends (Figure 5), correlated to the PEG content in the copolymer. The two copolymers with the largest fraction of PEG decreased their *θ*_adv_ of ~5° after 20 days of immersion. In water, the hydrolyzable silylester moieties were rapidly cleaved, leaving a carboxylate group in the main chain, thus increasing the copolymer hydrophilicity. At the same time, the more hydrophilic fraction was solubilized by exposing a fresh surface that was readily swelled by water with minor overall changes in the contact angle. For immersion times higher than 21 days, *θ*_adv_ remained constant for both the samples, consistently with the almost complete erosion of the films as observed from the mass loss measurements.

Conversely, PEG2-*b*-(TPSiMA30-*co*-MMA50), containing the lowest PEG content (20 mole%) showed the largest difference in *θ*_adv_ after prolonged immersion in water, with *θ*_adv_ dropping from 110° to 64° after 30 days of immersion. This is consistent with the slowest swelling and hydrolysis observed for this sample. In fact, for this film, a period of ~25 days in which the film slowly absorbed water without significant erosion was observed in the mass loss test, corresponding to the period in which the *θ*_adv_ decreased. Then, a small mass loss was observed; thus, after this time, the erosion and solubilization of the outermost hydrolyzed surface layers occurred over time until the original not-hydrolyzed copolymer surface was completely replenished, resulting in an increase in *θ*_adv_, reaching 95° after 75 days of immersion.

In any case, the receding contact angles (*θ*_rec_) were significantly lower (by 50°–80° at *t* = 0) than the corresponding *θ*_adv_, with consequently large hysteresis. Such a large hysteresis was consistent with a rapid change in the surface chemical composition of the films as a result of the hydrolysis of the silylester groups and the major exposure of the PEG chains at the polymer–water interface. *θ*_rec_ values also decreased as a function of immersion time following a trend similar to that already discussed for *θ*_adv_. Interestingly, PEG2-*b*-(TPSiMA28-*co*-MMA38) and PEG2-*b*-(TBSiMA26-*co*-MMA41), which had a comparable content of PEG but differed for the type of the silylester groups, showed a decrease in *θ*_rec_ of 2° and 11°, respectively, after 75 days of immersion, in agreement with the higher hydrolysis kinetics of TBSiMA with respect to TPSiMA.

### 3.5. Atomic Force Microscopy

Atomic force microscopy analysis was carried out to image the surface topography and evaluate the surface mechanical properties of the films PEG2-*b*-(TPSiMA30-*co*-MMA50), PEG2-*b*-(TBSiMA26-*co*-MMA41), and PEG2-*b*-(TPSiMA28-*co*-MMA38), either before or after immersion in ASW for 7 days to investigate possible changes in the surface structure of the films. In any case, AFM experiments were performed in air and immersed films were dried at room temperature for a few minutes.

**Figure 6:**
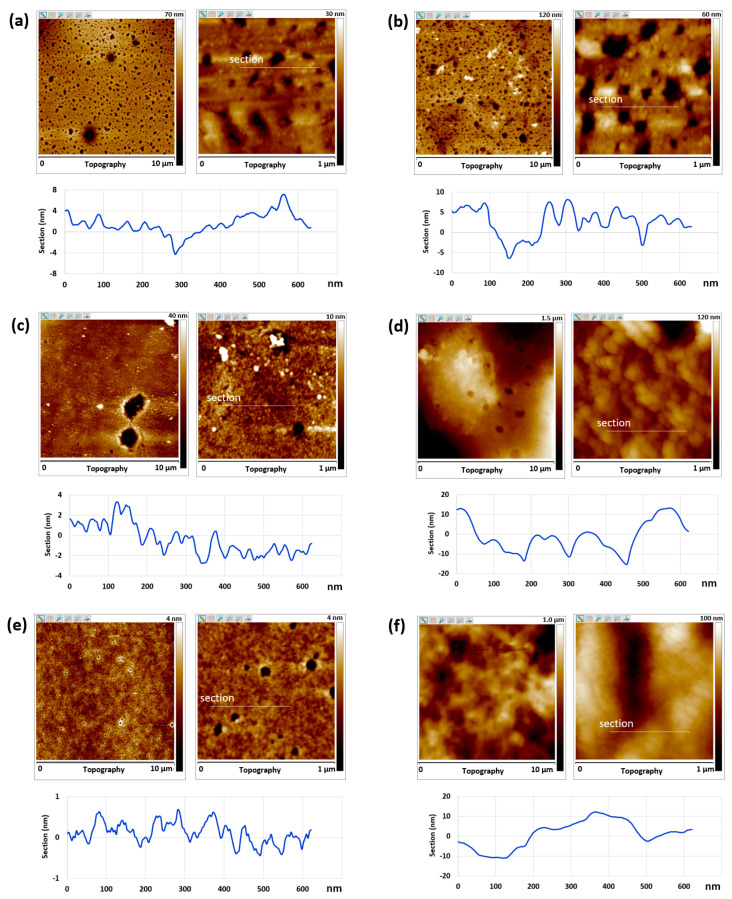
AFM topographic images (10 μm and 1 μm) and cross-section of the films before (**left**) and after (**right**) immersion in water for 7 days: PEG2-*b*-(TPSiMA30-*co*-MMA50) (**a**,**b**), PEG2-*b*-(TBSiMA26-*co*-MMA41) (**c**,**d**), PEG2-*b*-(TPSiMA28-*co*-MMA38) (**e**,**f**).

The topographical features on the film surfaces consisted of nano-domains (or granules) with size of 20–30 nm (Figure 6, left). The dimension of the nano-domains remarkably increased after immersion (60–100 nm). This phenomenon was again more evident for PEG2-*b*-(TBSiMA26-*co*-MMA41) and PEG2-*b*-(TPSiMA28-*co*-MMA38) surfaces as a result of their enhanced hydrophilicity after immersion in water and swelling of the PEG block (Figure 6, right). The hydrolysis of the silylester component might also play a role in the hydrophilization process of the film surface and its changes in topography.

**Figure 7:**
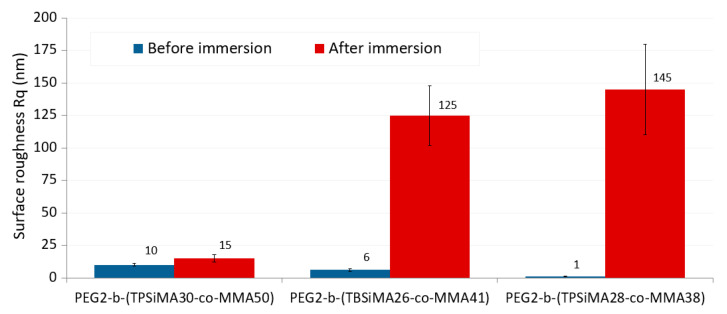
Surface roughness values calculated from the topographic images (10 μm × 10 μm) of PEG2-*b*-(TPSiMA30-*co*-MMA50), PEG2-*b*-(TBSiMA26-*co*-MMA41), and PEG2-*b*-(TPSiMA28-*co*-MMA38).

Before immersion, all the film surfaces appeared very smooth with root mean square values (*R*_q_) of 10 nm measured on areas of 10 μm × 10 μm (Figure 7). After immersion, the *R*_q_ values significantly increased, especially for films of PEG2-*b*-(TBSiMA26-*co*-MMA41) (*R*_q_ = 125 nm) and PEG2-*b*-(TPSiMA28-*co*-MMA38) (*R*_q_ = 145 nm) containing more PEG (33–34 mole%) than PEG2-*b*-(TPSiMA30-*co*-MMA50) (*R*_q_ = 15 nm) that displayed a more stable surface topography upon immersion in water (Figure 6). This result was a consequence of the surface erosion and/or film swelling in contact with ASW water.

Concerning the surface mechanical properties of the films, the AFM measurements did not reveal noticeable differences in the DMT elastic modulus of samples either before or after immersion (as shown in Figure S4 of the Supporting Materials). This result is not surprising since the characterization was performed on dried surfaces.

### 3.6. Biological Assay

**Figure 8:**
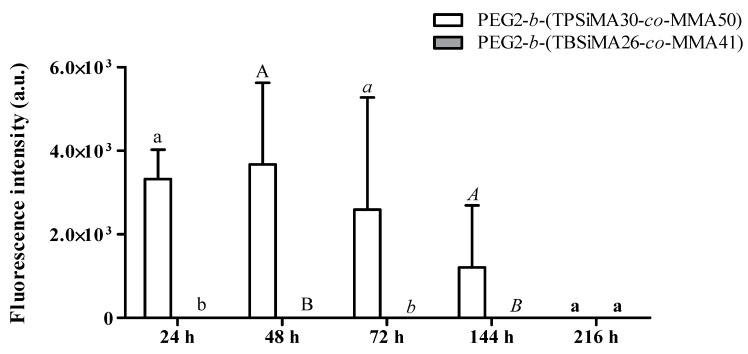
Intensity of fluorescence emitted by *Navicula salinicola* cells adherent to different surface samples after 24 h (lowercase letters), 48 h (uppercase letters), 72 h (italics lowercase letters), 144 h (italics uppercase letters), and 216 h (bold lowercase letters) of exposure. A PERMANOVA analysis (n = 4) was performed on sample results for each exposure time. Not-shared letters indicate statistically significant differences, *p* ≤ 0.05.

PEG2-*b*-(TBSiMA26-*co*-MMA41) and PEG2-*b*-(TPSiMA40-*co*-MMA50) films were selected for the biological assay with the diatom *Navicula salinicola*, given the different type of silylester and hydrolyzation kinetics, which was reflected in the distinct wettability behavior and surface topography. The settlement of diatoms was monitored after 24 h, 48 h, 72 h, 114 h (six days) and 216 h (nine days) (Figure 8). PEG2-*b*-(TBSiMA26-*co*-MMA41) with the highest hydrolysis rates completely inhibited the adhesion of *N. salinicola* cells at any investigated time. This result proves that the quick erosion of the film surface, as well as its changes in hydrophilicity, prevented the attachment of the cells. Conversely, PEG2-*b*-(TPSiMA30-*co*-MMA50) characterized by much slower hydrolysis kinetics displayed a non-zero value of intensity of fluorescence emitted by cells of *N. salinicola* adherent on the film after 24 h that, however, significantly decreased with time, reaching the zero value after nine days. This was due to the progressive erosion of the film surface that promoted the removal of the cells as the coating surface was renewed, according to the self-polishing mechanism.

## 4. Conclusions

PEGz-*b*-(TRSiMAx-*co*-MMAy) block copolymers were synthesized via RAFT polymerization and used to prepare self-supporting hydrolyzable films. The hydrolysis rate was found to be strongly affected by the type of trialkylsilyl group, with TBSiMA being much more susceptible to hydrolysis than TPSiMA. Moreover, longer chains of PEG, as well as higher amounts in the copolymers, were proven to increase the hydrolysis rate, while a higher concentration of MMA led to an opposite effect.

Dynamic contact angle measurements revealed that all the films underwent surface reconstruction as a result of two combined mechanisms, that is, the hydrolysis of the silyl ester bonds of the TRSiMA groups and the major exposure of the PEGMA chains in contact with the aqueous environment. The extent of each mechanism depended on the design of the copolymer, but both of them led to a hydrophilization of the film surface upon immersion in water. Surface modification of the film was also confirmed by AFM analysis, which proved an increase in surface roughness and nano-domain size upon immersion in ASW. The biological assay against the diatom *N. salinicola* demonstrated that films were able to completely prevent the diatom’s adhesion and proliferation. The timing for that strictly depended on the type of trialkylsilyl group, but in any case, did not exceed 9 days.

## Figures and Tables

**Table 1:** Reaction conditions for RAFT block copolymerization and characteristics of TRSiMA-based copolymers.

Copolymer	Mon:CTA ^a^	*p* ^b^	PEG/TRSiMA/MMA ^c^ (mole%)		M_n SEC_ ^d^ (g/mol)	*Ð* ^d^	TRSiMA ^e^ DP_n_	MMA ^e^ DP_n_
			Feed	Copolymer				
PEG2-*b*-(TPSiMA28-*co*-MMA38)	117:1	80	30/30/40	34/28/38	14,700	1.1	41	56
PEG2-*b*-(TPSiMA30-*co*-MMA50)	450:1	57	10/30/60	20/30/50	42,300	1.1	74	125
PEG1-*b*-(TPSiMA34-*co*-MMA39)	40:1	89	30/30/40	27/34/39	10,400	1.1	21	24
PEG1-*b*-(TPSiMA29-*co*-MMA60)	153:1	71	10/30/60	11/29/60	24,400	1.1	42	88
PEG2-*b*-(TBSiMA26-*co*-MMA41)	117:1	89	30/30/40	33/26/41	12,700	1.1	40	63
PEG2-*b*-(TBSiMA30-*co*-MMA54)	450:1	70	10/30/60	16/30/54	25,400	1.2	96	172
PEG1-*b*-(TBSiMA35-*co*-MMA37)	40:1	96	30/30/40	28/35/37	7100	1.2	22	23
PEG1-*b*-(TBSiMA24-*co*-MMA49)	153:1	92	10/30/60	27/24/49	5900	1.4	46	95
TPSiMA45-*co*-MMA55	58:1	86	0/40/60	0/45/55	7400	1.1	40	49
TBSiMA40-*co*-MMA60	64:1	96	0/40/60	0/40/60	6300	1.1	31	45

^a^ Monomers: CTA molar ratio in the feed. ^b^ Final conversion of methacrylic monomers determined via ^1^H NMR. ^c^ Molar percentage of PEG repeating units, TRSiMA (R = isopropyl, butyl) and MMA in the feed and copolymer. ^d^ Number-average molar mass and dispersity via SEC in chloroform. ^e^ Number-average degree of polymerization of TRSiMA and MMA repeating units in the second block, determined via ^1^H NMR.

**Table 2:** Values of advancing (*θ*_adv_)_,_ receding (*θ*_rec_) and hysteresis (Δ) for selected PEGz-*b*-(TRSiMAx-*co*-MMAy) copolymers as a function of immersion in artificial seawater.

Film			Days of Immersion		
		0	21	30	75
PEG2-*b*-(TPSiMA30-*co*-MMA50)	*θ*_adv_ (°)	110 ± 5	72 ± 6	64 ± 1	95 ± 4
	*θ*_rec_ (°)	30 ± 2	22 ± 2	23 ± 2	26 ± 2
	Δ (°)	80 ± 3	50 ± 5	41 ± 3	69 ± 2
PEG2-*b*-(TPSiMA28-*co*-MMA38)	*θ*_adv_ (°)	71 ± 3	66 ± 2	56 ± 1	57 ± 2
	*θ*_rec_ (°)	21 ± 2	19 ± 4	18 ± 1	19 ± 1
	Δ (°)	50 ± 3	47 ± 6	38 ± 1	38 ± 1
PEG2-*b*-(TBSiMA26-*co*-MMA41)	*θ*_adv_ (°)	82 ± 3	77 ± 6	68 ± 2	66 ± 5
	*θ*_rec_ (°)	28 ± 4	18 ± 2	18 ± 1	17 ± 4
	Δ (°)	54 ± 3	59 ± 4	50 ± 3	49 ± 2

## Data Availability

Not applicable.

## Supplementary Materials

The following supporting information can be downloaded at: https://www.mdpi.com/article/10.3390/polym14214589/s1, ^1^H NMR spectra, description of copolymer composition and *DP*_n_ calculation from ^1^H NMR spectra of PEG2-CTA and copolymers PEG2-*b*-(TBSiMA26-*co*-MMA41) and PEG2-*b*-(TPSiMA28-*co*-MMA38), description of the hydrolysis profile calculation from ^1^H NMR spectra, graphic of the surface elastic modulus of the films obtained by AFM measurements. Figure S1. ^1^H NMR spectrum of PEG2-CTA. ^1^H NMR (CDCl_3_, δ in ppm): 4.27 (COOCH_2_), 3.9–3.5 (CH_2_CH_2_O), 3.4 (OCH_3_), 3.35 (SCH_2_), 2.7 (CH_2_COO), 2.3–2.6 (CH_2_CH_2_COO), 1.9 (CH_3_CCN), 1.7 (SCH_2_CH_2_), 1.5–1.2 (CH_2_)_9_, 0.9 (CH_2_CH_3_). * Acetonitrile; Figure S2. ^1^H NMR spectrum of PEG2-*b*-(TBSiMA26-*co*-MMA41); Figure S3. ^1^H NMR spectrum of PEG2-^b^-(TPSiMA28-^co^-MMA38); Figure S4. Surface elastic modulus values of PEG2-*b*-(TPSiMA30-*co*-MMA50), PEG2-*b*-(TBSiMA26-*co*-MMA41), PEG2-*b*-(TPSiMA28-*co*-MMA38).
