# Supplementary material for: Polyethylene Glycol-b-poly(trialkylsilyl methacrylate-co-methyl methacrylate) Hydrolyzable Block Copolymers for Eco-Friendly Self-Polishing Marine Coatings
Source: Polymers (Basel). 2022 Oct 28;14(21):4589. doi: 10.3390/polym14214589 (PMC9656287; doi:10.3390/polym14214589)
Supplement: Supplementary file 1 [file polymers-14-04589-s001.zip › polymers-1993040-supplementary.pdf]

Supporting Information

Polyethylene Glycol-*b*-Poly(trialkylsilyl methacrylate-*co*-methyl methacrylate) Hydrolyzable Block Copolymers for Eco-Friendly Self-Polishing Marine Coatings

Elisa Guazzelli [1], Matteo Oliva [2], Carlo Pretti [2,3], Gianfranca Monni [3], Armand Fahs [4], Christine Bressy [4] and Elisa Martinelli [1,*]

[1] Dipartimento di Chimica e Chimica Industriale, Università di Pisa, 56124 Pisa, Italy
[2] Consorzio Interuniversitario di Biologia Marina e Ecologia Applicata "G. Bacci", 57128 Livorno, Italy
[3] Dipartimento di Scienze Veterinarie, Università di Pisa, 56124 Pisa, Italy
[4] Laboratoire MAPIEM, E.U.4323, SeaTech Ecole d'Ingénieurs, Université de Toulon, CS 60584, CEDEX 9, 83041 Toulon, France
* Correspondence: elisa.martinelli@unipi.it

Table of Contents

1. ^{1}H NMR spectra — page 2
2. Copolymer composition and DP_n calculation from 1HNMR spectra — page 4
3. Hydrolysis profile calculation from ^{1}H NMR spectra — page 5
4. Surface elastic modulus of the films — page 6

1. ¹H NMR spectra

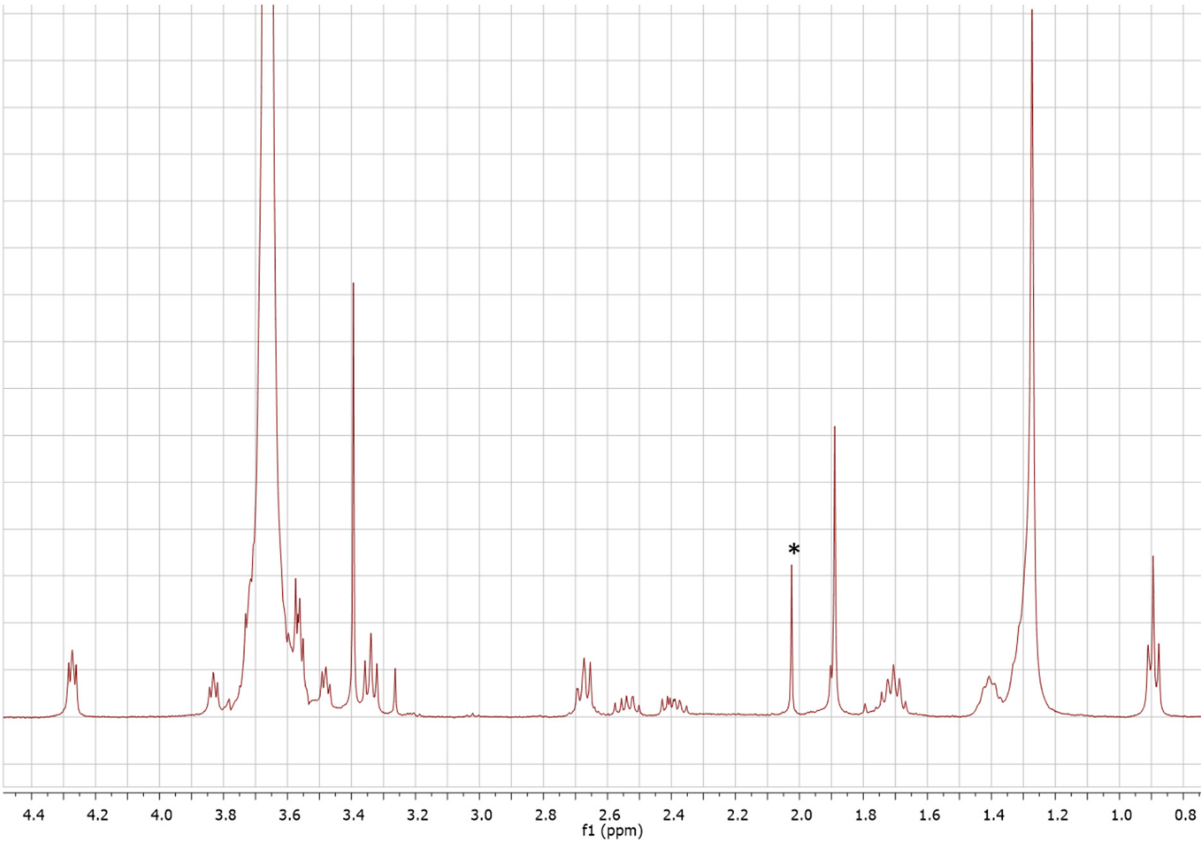

Figure S1. ^{1}H NMR spectrum of PEG2-CTA. ^{1}H NMR ($CDCl_3$, δ in ppm): 4.27 (COO**CH₂**), 3.9-3.5 (CH_2**CH₂**O), 3.4 (O**CH₃**), 3.35 (S**CH₂**), 2.7 (**CH₂**COO), 2.3-2.6 (**CH₂**CH_2COO), 1.9 (**CH₃**CCN), 1.7 (SCH_2**CH₂**), 1.5-1.2 (**CH₂**)$_9$, 0.9 (CH_2**CH₃**). * acetonitrile

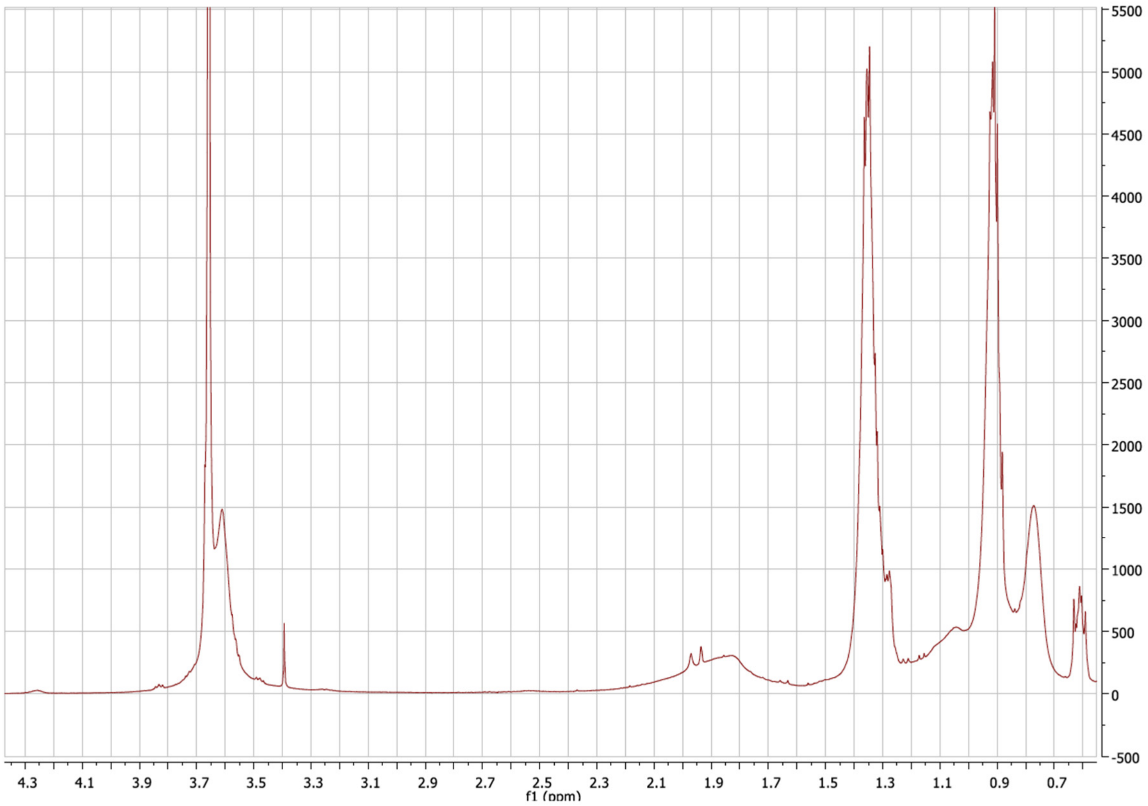

Figure S2. ^{1}H NMR spectrum of PEG2-*b*-(TBSiMA26-co-MMA41).

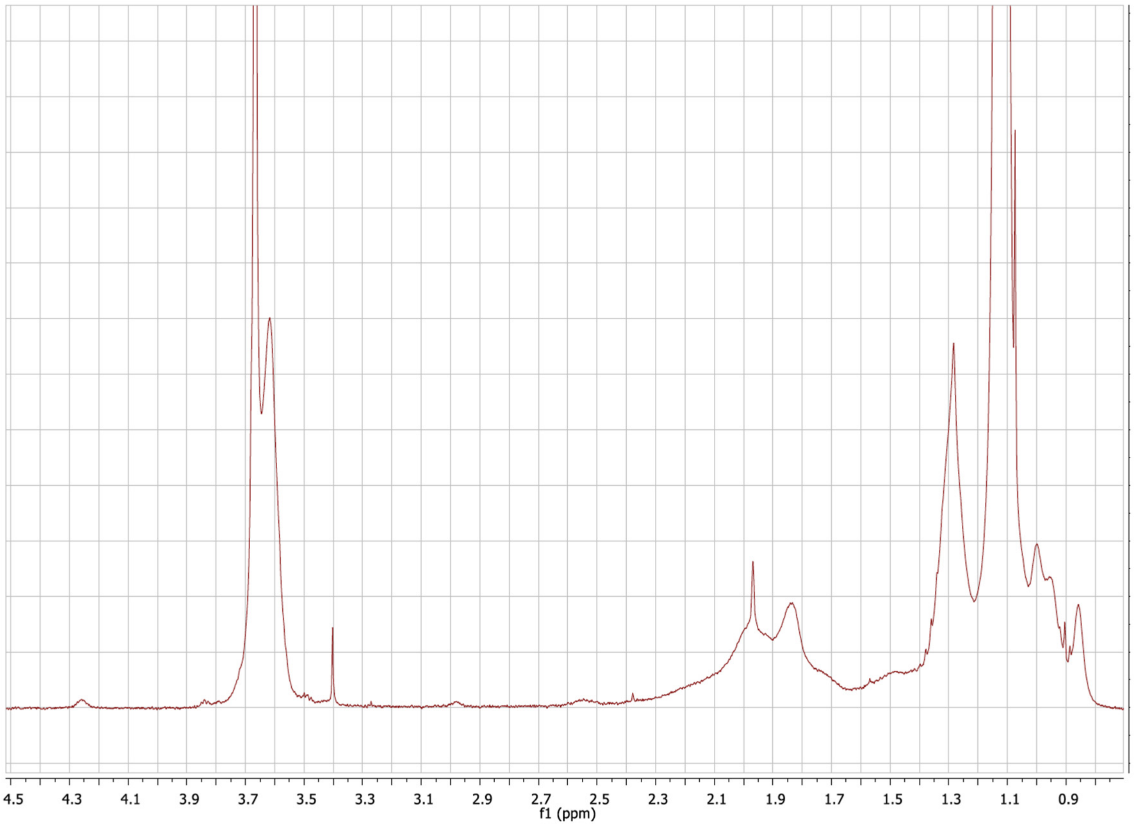

Figure S3. ^{1}H NMR spectrum of PEG2-*b*-(TPSiMA28-*co*-MMA38).

2. Copolymer composition and DP_n calculation from 1HNMR spectra

Molar composition of the purified copolymers was calculated from the number average degree of polymerization (DP_n) of each component of the copolymer. The DP_n of each component was estimated from ^{1}H NMR of the purified copolymers. Copolymer MMA molar content was determined by ^{1}H NMR taking as a reference the signal of the OCH_3 terminal of the PEG block at 3.48 ppm ($I_{3.48}$) and subtracting the contribution of OCH_2CH_2 of PEG (being $DP_{n\,PEG}$ = 17 or 50 for PEG1 and PEG2 respectively) repeating units superimposed on the signal at 3.8–3.5 ppm ($I_{3.8-3.5}$) of methyl protons ($COOCH_3$) of MMA as shown in Equation S1

$$DP_{n\,MMA} = \frac{I_{3.8-3.5} - 4\frac{I_{3.48}}{3} \times DP_{n\,PEG}}{3} \qquad \text{Equation S1}$$

The DP_n of TPSiMA was estimated by the difference from integrated area in the region 2.3–0.7 ppm ($I_{2.3-0.7}$) where all the protons from alkyl moieties of the silyl esters and the main methacrylic chain are superimposed (Equation S2).

$$DP_{n\,TPSiMA} = \frac{I_{2.3-0.7} - [5 \times DP_{n\,MMA} \times \frac{I_{3.48}}{3}]}{26} \qquad \text{Equation S2}$$

With a similar approach, the DP_n of TBSiMA was estimated by the difference from integrated area in the region 2.2–0.7 ppm ($I_{2.2-0.7}$) where all the protons from alkyl moieties of the silyl esters and the main methacrylic chain are superimposed (Equation S3)

$$DP_{n\,TBSiMA} = \frac{I_{2.2-0.7} - [5 \times DP_{n\,MMA} \times \frac{I_{3.48}}{3}]}{32} \qquad \text{Equation S3}$$

Finally, the molar percentage composition x and y of PEGz-*b*-(TRSiMAx-*co*-MMAy) copolymers was determined by ratio of the repeating units for each component, according to Eq. S1, Eq. S2, Eq. S3 and being $DP_{n\,PEG}$ = 17 or 50 (for PEG1 and PEG2 respectively) to the sum of the three that is the overall DP_n of the copolymer.

3. Hydrolysis profile calculation from 1HNMR spectra

The hydrolysis rate was studied in accelerated conditions by *in-situ* ^{1}H NMR by dissolving the copolymer in THF-d8 and adding a small aliquot of a pH 10 buffer to the solution. ^{1}H NMR spectra for the hydrolysis of PEG1-*b*-(TBSiMA24-co-MMA49) and PEG2-*b*-(TiPSiMA28-co-MMA38) are shown in Figure 3a and Figure 3b (main text). In both cases the signals of protons of the alkyl substituents in the silylester moieties shift to lower ppm passing from the repeating unit in the copolymers to the hydrolyzed siloxane product. Thus, the hydrolysis degree was estimated from Equation S1 for TBSiMA based copolymers as:

$$Hydrolysis\ degree\ (\%) \ = 100\ \frac{\frac{I_{0.5-0.6}}{6}}{\frac{I_{1.5-0.5}}{30+3\left(\frac{[MMA]}{[TBSiMA]}\right)}} \qquad \text{Equation S4}$$

where $I_{0.5\text{-}0.6}$ indicates the integrated area at 0.5–0.6 ppm and $I_{1.5\text{-}0.5}$ indicates the integrated area at 1.5 – 0.5 ppm (Figure 3 main text).

Similarly, the hydrolysis degree for TiPSiMA-based copolymers was estimated from Equation S2:

$$Hydrolysis\ degree\ (\%) \ = 100\ \frac{\frac{I^{t}_{1.1-0.92}-I^{t=0}_{1.1-0.92}}{18}}{\frac{I^{t}_{1.4-0.92}}{24+3\left(\frac{[MMA]}{[TiPSiMA]}\right)}} \qquad \text{Equation S5}$$

where $I^{t}_{1.4\text{-}0.92}$ and $I^{t}_{1.1\text{-}0.92}$ indicates the integrated area of the 1.4 – 0.92 and 1.1 – 0.92 ppm intervals, respectively, at different times. $I^{t=0}_{1.1\text{-}0.92}$ indicates the integrated area in the interval 1.1 – 0.92 ppm at the initial time, that was subtracted to take into account the superimposition of the signals from the main methacrylic chain that also fall in the region of interest (see Figure 3 main text).

4. Surface elastic modulus of the films

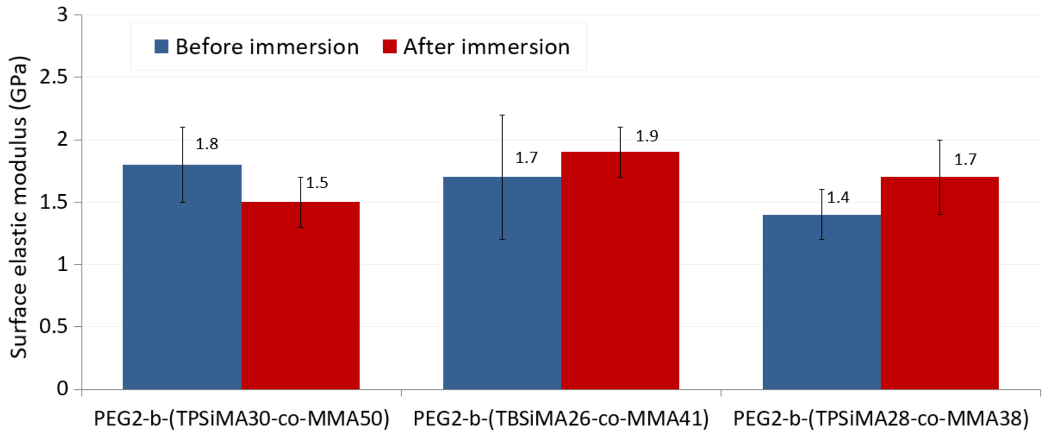

Figure S4. Surface elastic modulus values of PEG2-*b*-(TPSiMA30-*co*-MMA50), PEG2-*b*-(TBSiMA26-*co*-MMA41), PEG2-*b*-(TPSiMA28-*co*-MMA38).
